# The Rapid Antigen Detection Test for SARS-CoV-2 Underestimates the Identification of COVID-19 Positive Cases and Compromises the Diagnosis of the SARS-CoV-2 (K417N/T, E484K, and N501Y) Variants

**DOI:** 10.3389/fpubh.2021.780801

**Published:** 2022-01-03

**Authors:** Carlos Barrera-Avalos, Roberto Luraschi, Eva Vallejos-Vidal, Andrea Mella-Torres, Felipe Hernández, Maximiliano Figueroa, Claudia Rioseco, Daniel Valdés, Mónica Imarai, Claudio Acuña-Castillo, Felipe E. Reyes-López, Ana María Sandino

**Affiliations:** ^1^Centro de Biotecnología Acuícola, Facultad de Química y Biología, Universidad de Santiago de Chile, Santiago, Chile; ^2^Centro de Nanociencia y Nanotecnología CEDENNA, Universidad de Santiago de Chile, Santiago, Chile; ^3^Department of Biology, Faculty of Chemistry and Biology, University of Santiago de Chile, Santiago, Chile; ^4^Department of Cell Biology, Physiology and Immunology, Universitat Autònoma de Barcelona, Bellaterra, Spain; ^5^Facultad de Medicina Veterinaria y Agronomía, Universidad de Las Américas, Santiago, Chile

**Keywords:** rapid antigen test, SARS-CoV-2 detection, COVID-19 diagnosis, COVID-19 false negative rapid test for COVID-19 diagnosis, pandemic control strategies

## Abstract

Timely detection of severe acute respiratory syndrome due to coronavirus 2 (SARS-CoV-2) by reverse transcription quantitative polymerase chain reaction (RT-qPCR) has been the gold- strategy for identifying positive cases during the current pandemic. However, faster and less expensive methodologies are also applied for the massive diagnosis of COVID-19. In this way, the rapid antigen test (RAT) is widely used. However, it is necessary to evaluate its detection efficiency considering the current pandemic context with the circulation of new viral variants. In this study, we evaluated the sensitivity and specificity of RAT (SD BIOSENSOR, South Korea), widely used for testing and SARS-CoV-2 diagnosis in Santiago of Chile. The RAT showed a 90% (amplification range of 20 ≤ Cq <25) and 10% (amplification range of 25 ≤ Cq <30) of positive SARS-CoV-2 cases identified previously by RT-qPCR. Importantly, a 0% detection was obtained for samples within a Cq value>30. In SARS-CoV-2 variant detection, RAT had a 42.8% detection sensitivity in samples with RT-qPCR amplification range 20 ≤ Cq <25 containing the single nucleotide polymorphisms (SNP) K417N/T, N501Y and E484K, associated with beta or gamma SARS-CoV-2 variants. This study alerts for the special attention that must be paid for the use of RAT at a massive diagnosis level, especially in the current scenario of appearance of several new SARS-CoV-2 variants which could generate false negatives and the compromise of possible viral outbreaks.

## Introduction

The 2019 coronavirus disease (COVID-19) is a current pandemic respiratory disease caused by the severe acute respiratory syndrome coronavirus 2 (SARS-CoV-2) (1). In Chile, more than 1.6 million infections and 37 thousand deaths have been reported from this disease (2). Currently, the most effective way to prevent and control its spread is the timely detection and isolation of infected people. The reverse transcription quantitative PCR (RT-qPCR) is the recommended and widely used technique for diagnosis due to its high sensitivity and precision (3). However, new and faster methodologies for detecting and diagnosing have recently been implemented, including the rapid antigen test (RAT) for SARS-CoV-2. The technique's principle consists of a rapid chromatographic immunoassay for the qualitative detection of specific SARS-CoV-2 antigens from nasopharyngeal swab samples (NPSs) (4). This strategy has begun to be used in several countries, and its effectiveness compared to the standard RT-qPCR method has been the target of study and analysis (5). In Chile, it was implemented in Health centers by the Ministry of Health in March 2021 as an alternative to RT-qPCR. More than 20 million RT-qPCR tests and more than 750 thousand RATs have been carried out in Chile. Although its effectiveness has been verified in various studies showing differences between manufacturers, currently, there are no studies on the effectiveness of the antigenic tests used in Chile, nor on their performance for detecting the outbreak of new SARS-CoV-2 variants. In this study, we compare the efficacy of the results of 55 NPSs obtained by the rapid antigen test for SARS-CoV-2 (SD Biosensor, South Korea), a chromatographic assay using a monoclonal antibody against the nucleocapsid (N) protein of the SARS-CoV-2 virus, and the standard RT-qPCR detection method. We observed differences in the detection of positive COVID-19 cases for the different ranges of amplification assessed. Interestingly, we determined that this antigen test loses sensitivity for detecting SARS-CoV-2 variants carrying the K417N/T, E484K and N501Y single nucleotide polymorphisms (SNPs).

## Materials and Methods

### Samples

Nasopharyngeal swab samples (NPSs) of clinical patients included in this study were collected by the Primary Care Centers and the Hospitals that belong to the Central Metropolitan Health Service (Santiago of Chile) (SSMC, acronym in Spanish). The swab samples were taken, preserved, and transported using the CITOSWAB® transport kit (Cat. No. 2118-0015; Citotest Labware Manufacturing Co., Ltd, Jiangsu, China). All the samples arrived at the laboratory before the first 24 h after the sampling collection. These samples were processed in the laboratory of Virology (University of Santiago of Chile, USACH). Total RNA was extracted using the AccuPrep® Universal RNA Extraction Kit (Bionner, Daejeon, South Korea. Code product: K-3140) following the manufacturer's instructions. The extracted RNA was used immediately for RT-qPCR assays.

### SARS-CoV-2 Identification by Rapid Antigen Test (RAT), RT-qPCR, and Detection of Variants

The SARS-CoV-2 identification by rapid antigen test (SD BIOSENSOR, South Korea; Cat no: 99COV30D-ML02; Lot: QCO391081I) was made following the manufacturer's instructions. The viral SARS-CoV-2 genome sequence was carried out using the ORF1ab probe (TaqMan™ 2019nCoV Assay Kit v1; Thermo Fisher Scientific, Cat. No. A47532) following a one-step strategy. Positive internal control probes for ORF1ab and RNase P (TaqMan™ 2019-nCoV Control Kit v1; Thermo Fisher Scientific, Cat. No. A47533) were included and assessed individually in the 96-well PCR plate. The polymerase from TaqMan™ Fast Virus 1-Step Master Mix (Applied Biosystems™, Cat. No. 44-444-36) was included in each reaction. Each reaction contained 5 μl of TaqMan™ Fast Virus 1-Step Master Mix 4X, 1 μl of ORF1ab assay 20X (FAM detector channel), 1 μl of RNase P assay 20X (HEX detector channel), 11 μl of nuclease-free water, and 2 μl of extracted RNA sample. The thermal amplification conditions include the reverse transcription at 50 °C for 5 min, predenaturation at 95 °C for 20 s, followed by 40 cycles at 95 °C for 3 seconds and 60 °C for 30 seconds. All the RT-qPCR reactions were performed on the Agilent AriaMx Real-Time PCR System (Agilent Technologies, Part. No. G8830A). Data and graphics were extracted using the Agilent AriaMx software. The detection of different variants was made by the AccuPower® SARS-CoV-2 Variants ID Real-Time RT-PCR kit (Bioneer Cat. No. SMVR-2112) according to manufacturer instructions. The Exicycler 96 V4 Real-Time thermal cycler (Bioneer) was used for detecting fluorescence on the TET, TexasRed, FAM, TAMRA, and Cyanine5 channels. The data obtained were exported in an Excel spreadsheet, and the Cq value and relative fluorescence intensity were analyzed for the internal positive control, IPC (TAMRA), and each of the assessed variants.

### Ethics Statement

The experimental procedures included in this study were authorized by the Ethical Committee of the University of Santiago of Chile (No. 226/2021) and the Scientific Ethical Committee of the Central Metropolitan Health Service, Ministry of Health, Government of Chile (No. 370/2021), and following the Chilean law in force. Verbal consent for using of the sample for SARS-CoV-2 diagnostic purposes was given to the healthcare professional at CESFAM. Results were communicated to the patient in the Family Health Center they attended (CESFAM, acronym in Spanish; Central Metropolitan Health Service, Ministry of Health, Government of Chile).

## Results

**Figure 1:**
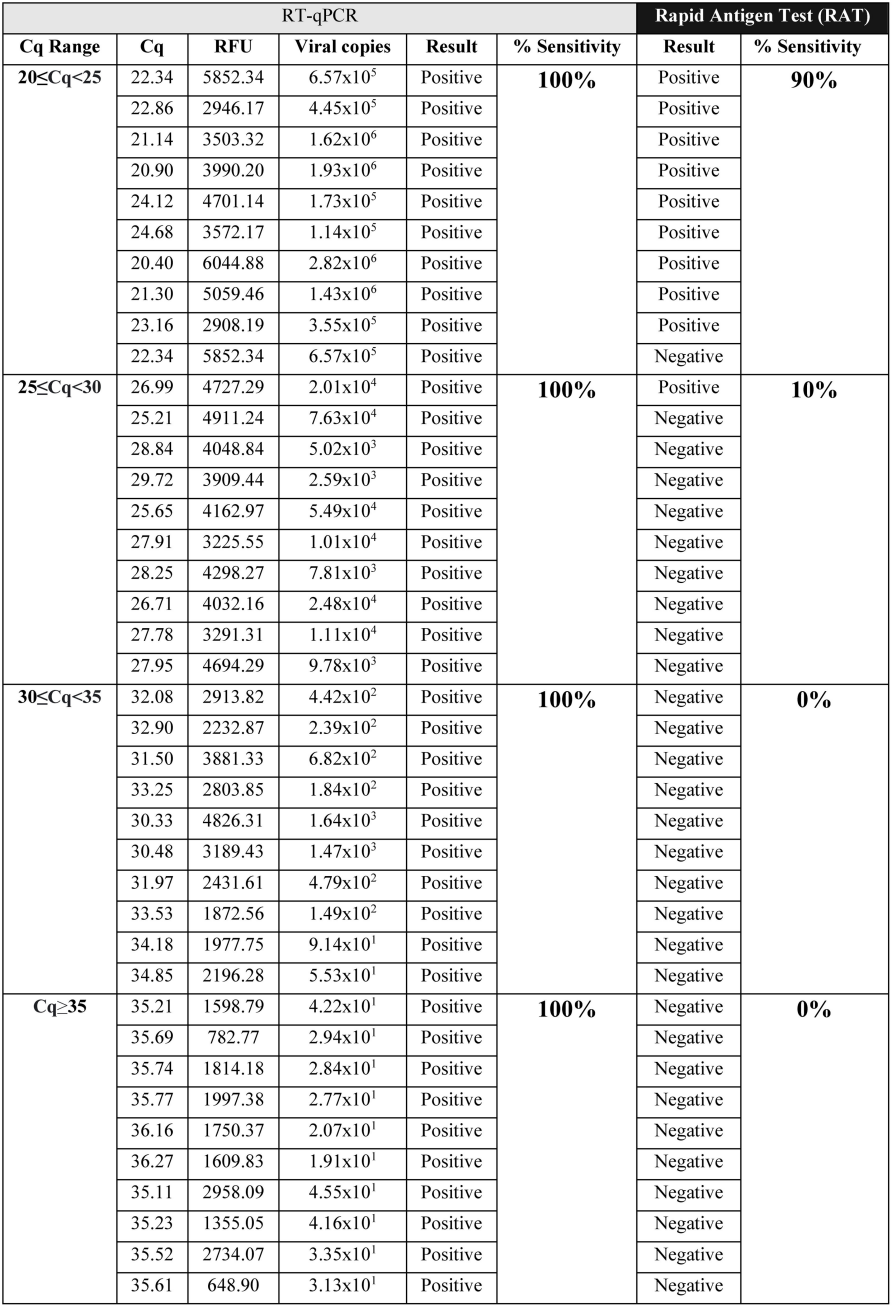
Sensitivity evaluation of the rapid antigen test (RAT; SD BIOSENSOR, South Korea) at different ranges of Cq values for ancestral strain of SARS-CoV-2 positive samples diagnosed by RT-qPCR. SARS-CoV-2 detection by RAT from RT-qPCR positive samples with Cq value between 20 ≤ Cq <25, 25 ≤ Cq <30, 30 ≤ Cq <35, and Cq≥35, respectively. All samples were positive by RT-qPCR. Table shows: the RFU (relative fluorescence units) and Cq value for the viral ORF1ab probe (*n* = 10 samples per Cq range).

To determine the detection sensitivity of the rapid antigen test (RAT), we evaluated 55 samples in different ranges of the cycle of quantification (Cq) for ORF1ab, in a similar way to studies previously reported (6, 7). The sensitivity of RAT was performed only for those samples containing the ancestral strain SARS-CoV-2 from Wuhan. Using NPSs, we determined that the detection sensitivity of RAT for the RT-qPCR Cq range 20 ≤ Cq <25 was 90% (Figure 1), while a 10% sensitivity was observed for samples comprised within the range of 25 ≤ Cq <30 (Figure 1). On the other hand, at higher Cq values, which include lower viral loads, the detection sensitivity was 0% for both Cq value ranges, 30 ≤ Cq <35 and Cq≥35 (Figure 1). We made semi-quantitative analysis of the RAT band intensity, where the SARS-CoV-2 positive samples for the Cq range 20 ≤ Cq <25 and 25 ≤ Cq <30 showed a high band intensity (Table 1). Negative NPSs samples were also determined. Altogether, data showed a significantly lower sensitivity of RAT for the detection of SARS-CoV-2 in comparison to RT-qPCR technique.

**Table 1:** Qualitative analysis of SARS-CoV-2 samples tested by Rapid Antigen Test (RAT).

	**Band intensity in RAT**		
**Cq ranges**	**None**	**Low**	**High**
20 ≤ Cq <25	1	0	9
25 ≤ Cq <30	9	0	1
30 ≤ Cq <35	10	0	0
Cq≥35	10	0	0

**Figure 2:**
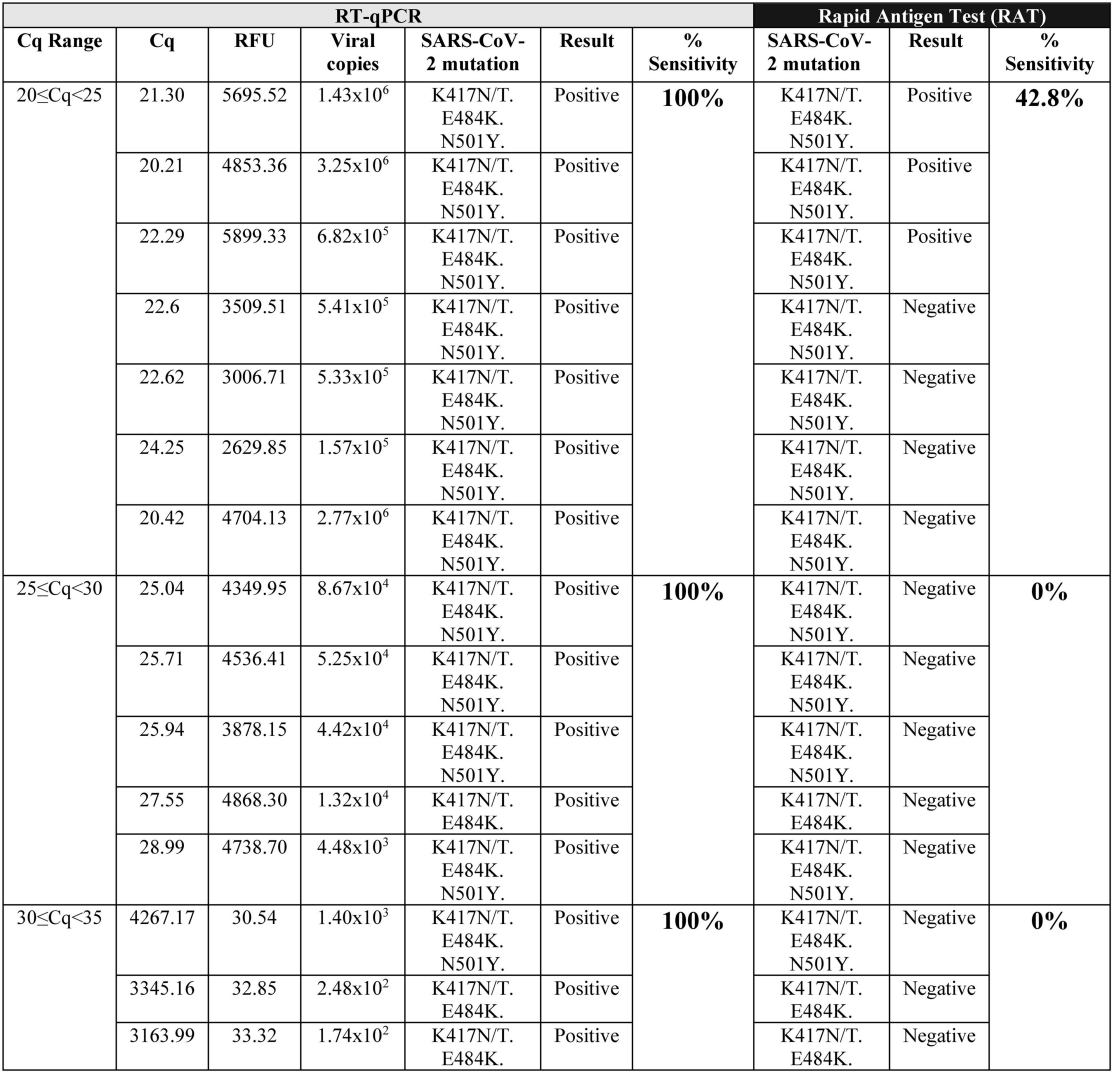
SARS-CoV-2 Variant detection by RAT. RAT detection of SARS-CoV-2 variants which contain K417N/T, E484K, and N501Y mutations, from RT-qPCR positive samples with Cq value between 20≥Cq <25, 25≥Cq <30, 30≥Cq <35. All samples were positive by RT-qPCR. Table shows: the RFU (relative fluorescence units) and Cq value for the viral ORF1ab probe (*n* = 16).

**Figure 3:**
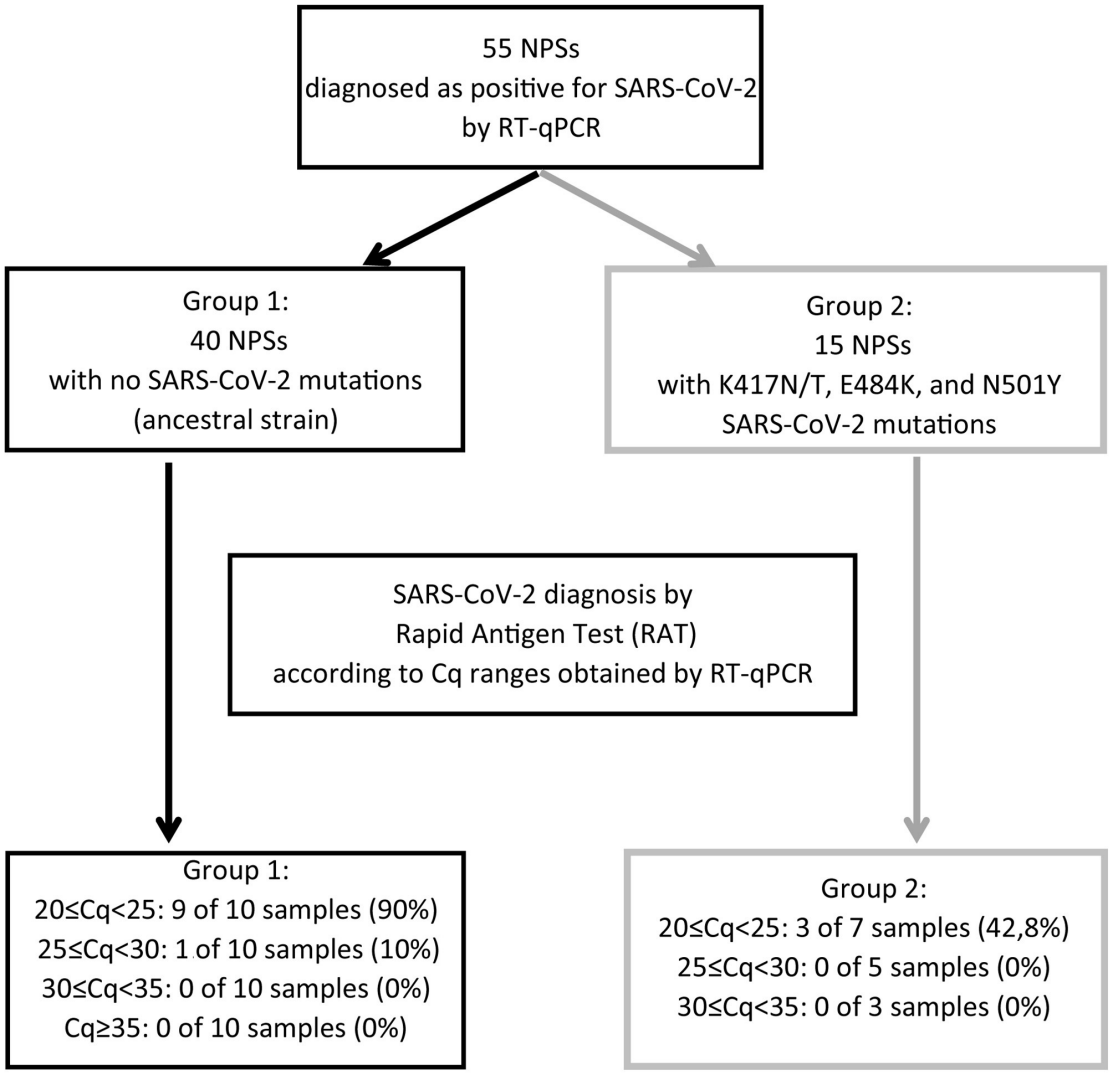
Summary of the results obtained for the analysis of the 55 nasopharyngeal swab samples (NPSs) evaluated by Rapid Antigen Test (RAT). The 55 NPSs were diagnosed as positive for SARS-CoV-2 using the ORF1ab probe following a one-step strategy. From them, 40 NPSs showed no mutations for SARS-CoV-2 (black arrow) (Group 1). By contrast, in the other 15 positive samples we identified the SARS-CoV-2 variants K417N/T, E484K, and N501Y (gray arrows) (Group 2). Each one of these groups were used to evaluate the sensitivity of the Rapid Antigen Test (RAT) according to the Cq ranges previously obtained by RT-qPCR. The group 1 showed a 90% of positive diagnosis using the RAT. However, the group 2 showed only a 42.8% of positive diagnosis. Importantly, all of them were grouped in the 20 ≤ Cq <25 interval.

On the other hand, we determined the ability of RAT to detect variants of the SARS-CoV-2 virus. We select NPSs from patients diagnosed as SARS-CoV-2 positive by RT-qPCR, containing variants according to the RT-qPCR SNP detection. When we evaluated these NPSs containing SARS-CoV-2 variants, we observed that the sensitivity of RAT decreased in the variants that carry K417N/T and E484K amino acid substitutions, even in samples with the lowest ranges of Cq (20 ≤ Cq <25). As a result, detection sensitivity decreases to 42% detection, obtaining 58% false-negative samples when the virus presents these mutations (Figure 2). Interestingly, from the three SARS-CoV-2 (K417N/T, E484K, and N501Y) positive samples for RAT, two of them presented a band of low intensity (Table 2) compared to SARS-CoV-2 samples with no mutations identified, which present a high-intensity band in the range 20≥Cq <25 (Table 1). Furthermore, for Cq over 25 no SARS-CoV-2 variant (K417N/T, E484K, and N501Y) sample was detected by RAT. A flow chart of the results obtained are described on Figure 3.

**Table 2:** Qualitative analysis of SARS-CoV-2 variant (K417N/T/, E484K, and N501Y) samples tested by Rapid Antigen Test (RAT).

	**Band intensity in RAT**		
**Cq Ranges**	**None**	**Low**	**High**
20 ≤ Cq <25	4	2	1
25 ≤ Cq <30	5	0	0
30 ≤ Cq <35	3	0	0

Taking together, these data suggest that RAT loses its full ability to detect SARS-CoV-2 against these variants.

## Discussion

RT-qPCR test is the gold standard technique for COVID-19 diagnosis (3). However, despite its high sensitivity, the extent of analysis and the associated costs have encouraged the use of faster and low-cost SARS-CoV-2 detection assays for better control of the pandemic (8). Previous studies have documented the efficiency of different brands of rapid antigen tests for SARS-CoV-2 detection as a diagnostic alternative compared to standard RT-qPCR. For example, Chaimayo et al. compared the sensitivity and specificity of the Standard Q COVID-19 Ag rapid test (SD Biosensor) against Allplex ™ 2019-nCoV Assay RT-qPCR assay (Seegene) in the diagnosis of COVID-19 in a Thailand population (9). They showed a 98% of comparable sensitivity with the real-time RT-PCR assay. Other rapid detection kits have shown, for example, a sensitivity of 85% (Shenzhen Bioeasy Biotech-2019-nCoV Ag) (10) or 72% (Abbott-Panbio COVID-19 Ag) (11), while the minimum sensitivity accepted in the rapid test by the World Health Organization is 80% for its diagnostic use (12). Dinnes et al. (5), after an analysis of 58 rapid tests, concluded that sensitivity of antigen assays significantly decrease in asymptomatic patients or after the second week of infection and, indicated that the sensitivity of these tests increases with the Cq ≤ 25. For example, Linares et al. (13) reported that patients with less than seven days of symptoms showed a high viral load and a sensitivity of 86.5%, while the sensitivity dropped to 53.8% in asymptomatic patients or lower viral loads using the Panbio COVID-19 Ag Rapid Test Device (Abbot). The same effect was observed using the SD Biosensor-STANDARD Q COVID-19 test, where a sensitivity of 65.3% was observed in symptomatic patients and 44% in asymptomatic patients (14). Krüttgen et al. (6) using the rapid SARS-CoV-2 antigen test from the Roche manufacturer, indicated that the sensitivity of the assay in 75 NPSs is 100% (Cq ≤ 25), 95% (Cq ≤ 30), 44.8% (Cq ≤ 35), and 22.2% (Cq>35) compared to RT-qPCR. Such sensitivity is directly related to the patient's viral load. In the same line, Schildgen et al. (15) reported a sensitivity of 100% in symptomatic patients and 84.6% in asymptomatic patients using the rapid test from Roche.

In this study, we observed that the rapid antigen test (SD Biosensor) has a detection efficiency depending on the Cq values, being 90, 10, 0, and 0% within the ranges of 20 ≤ Cq <25; 25 ≤ Cq <30; 30 ≤ Cq <35, and Cq≥35, respectively. In this regard, the manufacturer and distributor indicate that 95.5% of effectiveness at Cq <30. Although our study shows lower sensitivity as previously described, it is also important to mention that we used a smaller number of samples in each Cq range compared to previous evidence (16). Furthermore, we observed that RAT sensitivity for detecting ancestral strain of SARS-CoV-2 decreased from 90 to 42.8% with the SARS-CoV-2 variants that carry K417N/T, E484K and N501Y amino acid substitutions, even in the lower Cq range of 20 ≤ Cq <25. The low intensity of bands showed for two of the three SARS-CoV-2 variants identified by RAT suggests a decreased specificity for samples with the K417N/T, E484K, and N501Y mutations. By contrast, the virus with no mutations showed a high intensity band of detection by RAT in the 90% of the samples evaluated. Because the amino acid substitutions E484K and N501Y are present in the beta (B.1.351) and gamma (P.1) variants, and the substitution K417N is found in the beta variant and K417T in the gamma variant (17), we hypothesized that RAT might not detect both or at least one of these two virus variants. In this line, Frediani et al. (7) reported that the Abbott BinaxNOW COVID-19 Ag Card rapid test detects the P.1 variant, even between the Cq ranges of 20-22. However, this data corresponds only to one sample analyzed (7). Other variant studies using rapid antigen tests, including analysis of the alpha (B.1.1.7) and beta (B.1.351) variants (18), showed no detection at Cq values between 29–35 (7) like this study.

The rapid antigen test analyzed in this study detects the nucleocapsid (N) of the original SARS-CoV-2 virus using a monoclonal antibody. This kind of detection can explain the low capacity of RAT to detect the variants, which has the substitutions P80R and R203K in the N protein. These data further suggest that this rapid antigen test could also fail to detect other variants, such as delta (B.1.617.2), because, in addition of Spike protein mutations (19), the delta variant also has mutations in the N protein (different to R203K). This data suggests a lack of specificity of RAT to detect specific variants, which agrees with the fact that RAT initially manufactured to detect the ancestral strain of SARS-CoV-2. In addition, this takes on real relevance when the RAT is used as an active search or mass testing in public places in infected patients with a high Cq (low viral load). According to the results shown in our work, the diagnosis by RAT will be negative, and the patient will be able to infect their contacts and, consequently, may generate important local outbreaks. Therefore, detecting positive patients with low viral load, who will probably be asymptomatic, should be done only by the RT-qPCR assay, one of the safest ways to be massively tested in the population to control the pandemic. Further studies are needed for evaluating by RAT a higher number of samples and SARS-CoV-2 variants. Although we found significant differences in each condition (ancestral strain of SARS-CoV-2; SARS-CoV-2 variants), it is still necessary to increase the number of samples to obtain more accurate detection percentages of SARS-CoV-2 in each range of Cq analyzed. We suggest restricting the use of RAT only to confirm COVID-19 symptomatic positive cases but not as a massive strategy for traceability and identification of cases in the population.

## Conclusions

The sensitivity of the rapid antigen test (SD Biosensor, South Korea) is determined by the viral load of the sample. While high viral loads (20 ≤ Cq <25) present 90% sensitivity, 25 ≤ Cq <30 and >30 present 10 and 0% respectively. On the other hand, the rapid test reduces its detection sensitivity compared to samples positive for SARS-CoV-2, but that present the mutations K417N/T, E484K, and N501Y observing a detection capacity of 42% in ranges of 20 ≤ Cq <25.

## Data Availability Statement

The original contributions presented in the study are included in the article/supplementary material, further inquiries can be directed to the corresponding author/s.

## Ethics Statement

The studies involving human participants were reviewed and approved by Ethical Committee of the University of Santiago of Chile (No. 226/2021), and Scientific Ethical Committee of the Central Metropolitan Health Service, Ministry of Health, Government of Chile (No. 370/2021). Written informed consent for participation was not required for this study in accordance with the national legislation and the institutional requirements.

## Author Contributions

CB-A, RL, MI, CA-C, FER-L, and AMS: conceptualization. CB-A, RL, and FER-L: data curation. CB-A, RL, FER-L, and AMS: formal analysis. MI, CA-C, FER-L, and AMS: funding acquisition. CB-A, AM-T, FH, MF, and CR: investigation. CB-A, EV-V, FER-L, and AMS: methodology. DV, FER-L, and AMS: supervision. DV, MI, CA-C, FER-L, and AMS: validation. CB-A, RL, CA-C, and FER-L: writing—original draft. CB-A, RL, EV-V, CA-C, MI, FER-L, and AMS: writing—review and editing. All authors have read and agreed to the published version of the manuscript.

## Funding

The Laboratory of Virology supported the COVID-19 diagnosis in the University laboratories network (Ministry of Sciences, Ministry of Health, Government of Chile) for diagnosis tasks. The funders had no role in study design, data collection and analysis, decision to publish, or manuscript preparation.

## Conflict of Interest

The authors declare that the research was conducted in the absence of any commercial or financial relationships that could be construed as a potential conflict of interest.

## Publisher's Note

All claims expressed in this article are solely those of the authors and do not necessarily represent those of their affiliated organizations, or those of the publisher, the editors and the reviewers. Any product that may be evaluated in this article, or claim that may be made by its manufacturer, is not guaranteed or endorsed by the publisher.
